# Patient-centered care during the last year of life: adaptation and validation of the German PACIC short form for bereaved persons as proxies (PACIC-S9-proxy)

**DOI:** 10.1186/s12904-020-00687-x

**Published:** 2020-11-24

**Authors:** Vera Vennedey, Gloria Dust, Nicolas Schippel, Arim Shukri, Julia Strupp, Christian Rietz, Raymond Voltz, Stephanie Stock

**Affiliations:** 1 Institute for Health Economics and Clinical Epidemiology, Gleueler Straße 176-178, 50935 Cologne, Germany; 2 Department of Palliative Medicine, Medical Faculty, University of Cologne, Cologne, Germany; 3 Institute of Medical Sociology, Health Services Research, and Rehabilitation Science (IMVR), University of Cologne, Cologne, Germany; 4 Heidelberg University of Education, Heidelberg, Germany; 5 Center for Health Services Research Cologne (ZVFK), University of Cologne, Cologne, Germany; 6 Center for Integrated Oncology Aachen Bonn Cologne Düsseldorf (CIO ABCD), University of Cologne, Cologne, Germany; 7 Clinical Trials Center Cologne (ZKS), University of Cologne, Cologne, Germany

**Keywords:** Last year of life, End-of-life, Patient-centered care, Patient-centered care, Questionnaire, Cross sectional study, Validity, Factor analysis, PACIC

## Abstract

**Background:**

Providing patient-centered care (PCC) during the last year of life (LYOL) can be challenging due to the complexity of the patients’ medical, social and psychological needs, especially in case of chronic illnesses. Assessing PCC can be helpful in identifying areas for improvements. Since not all patients can be surveyed, a questionnaire for proxy informants was developed in order to retrospectively assess patient-centeredness in care during the whole LYOL. This study aimed to evaluate the feasibility and validity of an adapted version of the German Patient Assessment of Chronic Illness Care (PACIC) for surveying bereaved persons in order to assess PCC during the decedents’ LYOL.

**Methods:**

The German PACIC short form (11 items) was adapted to a nine-item version for surveying bereaved persons on the decedent’s LYOL (PACIC-S9-Proxy). Items were rated on a five-point Likert scale. The PACIC adaptation and validation was part of a cross-sectional survey in the region of Cologne. Participants were recruited through self-selection and active recruitment by practice partners. Sociodemographic characteristics and missing data were analyzed using descriptive statistics. An exploratory factor analysis was conducted in order to assess the structure of the PACIC-S9-Proxy. Internal consistency was estimated using Cronbach’s alpha.

**Results:**

Of the 351 informants who participated in the survey, 230 (65.52%) considered their decedent to have suffered from chronic illness prior to death. 193 of these informants (83.91%) completed ≥5 items of the questionnaire and were included in the analysis. The least answered item was item (74.09%) was item 4 (encouragement to group & classes for coping). The most frequently answered item (96.89%) was item 2 (satisfaction with care organization). Informants rated the item” Given a copy of their treatment plan” highest (mean 3.96), whereas “encouragement to get to a specific group or class to cope with the condition” (mean 1.74) was rated lowest. Cronbach’s alpha was 0.84. A unidimensional structure of the questionnaire was found (Kaiser-Meyer-Olkin 0.86, Bartlett’s test for sphericity *p* < 0.001), with items’ factor loadings ranging from 0.46 to 0.82.

**Conclusions:**

The nine-item questionnaire can be used as efficient tool for assessing PCC during the LYOL retrospectively and by proxies.

**Trial registration:**

The study was registered in the German Clinical Trials Register (DRKS00011925) on 13 June 2017.

**Supplementary Information:**

The online version contains supplementary material available at 10.1186/s12904-020-00687-x.

## Background

Care for chronically ill multimorbid patients is a major challenge for many healthcare systems. Especially during the last year of life (LYOL) patients experience a high burden of physical symptoms and psychosocial needs which need to be addressed. At the same time, the overall use of healthcare services usually increases during the LYOL leading to multiple transitions between care settings [1]. (e.g. home to hospital; from one specialist to another, hospital to nursing home) [2, 3]. If these transitions are not managed properly, patients will experience disruptions and failures in coordination of care, which, at the worst-case scenario, can lead to preventable readmissions and patient burden. Patients and their relatives therefore expect that end-of-life care to be characterized by a patient-centered approach in the sense described by Balint as considering a patient as a “unique human-being” [4] (p. 269) instead of regarding them purely as an illness to treat. Later, the Institute of Medicine (IOM) established the widely accepted definition of PCC as “care that is respectful of and responsive to individual patient preferences, needs, and values and ensuring that patient values guide all clinical decisions” (p. 5) [5]. Patient-centeredness in care is also considered a core attribute of palliative care, being reflected in the second guiding principle of the German Charter of Care for severely ill and dying persons in Germany [6]. Important features of PCC during the LYOL include informing patients about their options and treatments, involving them in care, coordinating care, delivering interdisciplinary team based care, and providing physical and emotional support [7–14]. PCC thus aims to support patients’ autonomy and their active involvement in the choice of treatment, and to alleviate caregiver burden. Several studies report positive associations between patient-centeredness in care for elderly, multimorbid or palliative patients and their health outcomes and/or satisfaction with their care [15, 16].

Patients are often severely ill during their LYOL and suffer from physical or cognitive constraints, which sometimes make it difficult to ask the patients themselves about their needs, values and preferences for care are. As such, clinicians and researchers often have to rely on the opinions of relatives, friends or other contact persons with a personal or occupational attachment to the patient. Additionally, as this study aimed to assess the patient-centeredness throughout the whole LYOL, retrospectively surveying friends and relatives was necessary. Several studies used proxy informant studies and investigated the feasibility of proxy-informants on topics related to the LYOL [17, 18]. A review of such studies found proxy answers to be reliable in the context of quality of healthcare services and more objective reporting of symptoms [19]. The available studies mainly focus on experiences during the LYOL in specific settings such as in long-term or palliative care setting, acute care or the quality of care in general [17, 20]. None of the instruments specifically addresses patient-centeredness during the LYOL. One survey instrument commonly used to assess patient-centeredness is the Patient Assessment of Chronic Illness Care (PACIC) [20]. However, this instrument is only validated for surveying patients. As the LYOL can only be determined retrospectively, it is necessary to survey proxy informants is necessary. To the best of our knowledge, none of the questionnaires currently available can be used to retrospectively assess the level of patient-centeredness of care during the LYOL. This study aimed to evaluate the feasibility and validity of an adapted PACIC short form in surveying bereaved persons regarding the perception of patient-centeredness in the care of the decedents.

## Methods

### Setting

This project was conducted within the Cologne Research and Development Network (CoRe-Net), which consists of scientists, patient organizations, HSCOs, municipality representatives and other stakeholders. The data collection for this study took place within the research project LYOL-C (Last Year of Life - Cologne), which is one of three initial projects affiliated with CoRe-Net [21, 22]. CoRe-Net members participated in developing ideas on the conducting of the study. A multi-item and multi-scale questionnaire for bereaved persons was set up to assess the process and experience of care during the LYOL of the deceased person. The questionnaire included socio-demographic questions related to the informant and decedent, a chronological survey of places of care within the LYOL, wishes and experiences of in the LYOL, a recently developed German version of the VOICES [23], and a newly adapted version of the German PACIC short form [24] – named the PACIC-S9-Proxy. The original PACIC and its short form’s psychometric properties have been analyzed previously [24–26]. This paper reports on the adaptation and validation of the PACIC-S9-Proxy, and the level of patient-centeredness experienced in the LYOL. The results of the VOICES data are presented elsewhere [27].

### Questionnaire adaptation

For this study, the German short version of the PACIC was adapted [24–26]. First, the answer scale of the PACIC short form (percentages) was switched back to the format of the answer scale from the original German PACIC long version. This scale is easier to understand, since answers can be provided on a five-point scale corresponding to “almost never” (=1), “generally not”, “sometimes”, “most of the time“, and “almost always” (=5). To prevent the informants from choosing random answers, the answer category “unable to rate” was included. This category ensures that only those informants who feel able to provide valid data are included [28]. This answer category also facilitates an analysis of which aspects informants might be less able to provide information for with regard to patient-centeredness. As a second adaptation, items three (being supported in setting goals for healthier lifestyle) and six (being asked about health behaviour e.g. smoking) of the German PACIC short form were excluded since they were not deemed particularly important in care during the LYOL. Lastly, the phrasing of items was adapted to address the bereaved relatives rather than the decedents e.g. the item “How often was I satisfied that my care was well organized?” was adapted to “How often was your loved one satisfied that his / her care was well organized?” The adapted version was deemed comprehensible and feasible in eight cognitive pre-tests involving persons from the target group and no further adaptation appeared to be necessary. An English version of the questionnaire is provided in supplementary file 1. The original German questionnaire is available upon request.

### Participants, recruitment & data collection

Informants were eligible for study participation if they had lost a person close to them (e.g. friend, relative or partner) within the last 12 months, and if this deceased person lived in the region of Cologne and did not die due to an accident or killing. Both, the study participant and the decedent had to be at least 18 years old. For data protection reasons, the eligibility criteria were self-assessed and not verified by registry offices or similar bodies. Around 100 practice partners participated in the recruitment, including general practitioners, nursing homes, outpatient nursing services, hospitals, working groups in palliative and hospice care, morticians, health insurance companies, self-help groups, churches, public health services, community colleges, municipal representatives for senior citizens in Cologne, and civic centers. Two strategies were used for the recruitment of participants: i) Direct distribution of questionnaires by cooperating practice partners, either in person or by mail using the details in the patients’/ clients’ records. ii) Self-selection via newspaper articles or flyers and posters, which were also placed in the practice partners’ institutions. Potential participants were asked to contact the study team to receive a questionnaire by regular mail. People who requested a questionnaire, but did not return it received one reminder. Data were collected between November 2017 and August 2018 in a cross sectional study.

### Data analysis

All analyses were conducted using IBM SPSS Statistics for Windows, version 25 (IBM Corp., Armonk, N.Y., USA). All data was pseudonymized for the analyses. Socio-demographic data were analysed using descriptive statistics. All returned PACIC-S9-Proxy questionnaires with at least five out of nine items from completed with an answer category ranging from “almost never” (=1) to “almost always” (=5) were included in the analysis. Means and standard deviations were calculated for each item. Items which participants felt unable to rate and missing values were imputed using the mean values for the respective items from the overall sample. As in the original PACIC versions, the answer scale was converted during the analysis into a cardinal scale, where 1 corresponds to the worst rating of an item and 5 to the best rating. The internal consistency of the questionnaire was estimated using Cronbach’s alpha, which can range from 0 to 1 where 0 corresponds to no correlation among the items and 1 to perfect correlation. An exploratory factor analysis was performed using principal factor analysis (eigenvalue > 1, varimax rotation). The Kaiser-Meyer-Olkin (KMO) criterion of sampling adequacy was used and Bartlett’s test of sphericity was conducted. An alpha level of *P* < 0.05 was applied in significance tests. In sensitivity analyses only questionnaires in which all nine items had been answered were considered.

## Results

### Participant characteristics

Out of the 351 informants who answered the questionnaire, 230 (65.53%) stated that the deceased person had suffered from a chronic illness. Of this number, 193 (83.91%) felt able to provide rating and completed at least five items of the PACIC-S9-Proxy with an answer ranging from “almost never” (=1) to “almost always” (=5), and were therefore included in the analysis. The majority of the informants were spouses (44.04%) or children (40.41%) of the decedent, and had an average age of 69 (range: 22–87). The decedents were 79 years on average and the majority suffered from oncological conditions (60.10%) and various cardiovascular diseases (51.81%). Further socio-demographic characteristics can be found in Table 1.

**Table 1:** Informant and decedent characteristics (*N* = 193)

	Characteristic	n	%
**Informant**	**Age (average 69 years)**		
	18–29 years	1	(0.52)
	30–49 years	26	(13.47)
	50–64 years	91	(47.15)
	65–79 years	63	(32.64)
	80+ years	12	(6.22)
	**Sex**		
	Male	45	(23.31)
	Female	148	(76.68)
	**Relationship to decedent**		
	Spouse	85	(44.04)
	Son/daughter	78	(40.41)
	Sibling	6	(3.11)
	Son-in-law/daughter-in-law	3	(1.55)
	Parent	2	(1.04)
	Other relationship	7	(3.63)
	Friend	8	(4.15)
	Other	4	(2.07)
**Decedent**	**Age at death (average 79 years)**		
	18–29 years	1	(0.51)
	30–49 years	2	(1.04)
	50–64 years	44	(22.80)
	65–79 years	62	(32.12)
	80+ years	84	(43.52)
	**Sex**		
	Male	96	(49.74)
	Female	97	(50.26)
	**German citizenship**		
	Yes	187	(96.89)
	No	6	(3.11)
	**Family situation**^**a**^		
	Had a partner	94	(48.7)
	Lived together with partner	70	(36.27)
	Had children	97	(50.26)
	Lived together with children	20	(10.36)
	Lived together with someone else	11	(5.70)
	Lived alone	64	(33.16)
	**Illnesses in the last year of life**^**a**^		
	Cancer	116	(60.10)
	Cardiovascular disease	100	(51.81)
	Neuro-psychological disease	93	(48.19)
	Disease of the respiratory system	89	(46.11)
	Liver or kidney disease	39	(20.21)
	Diabetes mellitus	31	(16.06)
	Other	54	(27.98)
	^a^ Multiple responses were possible.		

### Results of questionnaire evaluation

The mean ratings and distributions of items are displayed in Table 2. On average, informants rated item 3 (given medication plan) highest (3.69 of 5). In contrast, informants stated that the decedent was “generally not” (1.74 of 5, Item 4) encouraged to seek groups or classes for coping with the illness. This item was also the item for which informants most often considered themselves unable to provide a rating (24.4%). Item 2 (satisfaction with well-organized care) was answered most often (96.1%). Informants stated that on average decedents were moderately to rather satisfied (3.52 out of 5) with the medical care they received.

**Table 2:** Item frequencies & means (*N* = 193)

Item What was your relative’s experience of the care they received for their chronic illness during the last year of life?		almost never n (%)	generally not n (%)	sometimes n (%)	most of the time n (%)	almost always n (%)	unable to rate n (%)	Missing n (%)	Mean rating^**a**^	SD
1	… were they given choices about treatment to think about.	47 (24.35)	26 (13.47)	27 (13.99)	29 (15.03)	40 (20.73)	20 (10.36)	4 (2.07)	**2.94**	1.45
2	… were they satisfied that their care was well organized.	10 (5.18)	25 (12.95)	14 (7.25)	75 (38.86)	63 (32.64)	6 (3.11)		**3.83**	1.17
3	… were they given a copy of their treatment plan.	30 (15.54)	12 (6.22)	7 (3.63)	22 (11.40)	113 (58.55)	9 (4.66)		**3.96**	1.51
4	… were they encouraged to get to a specific group or class to help them cope with their chronic condition.	101 (52.33)	11 (5.70)	11 (5.70)	7 (3.63)	13 (6.74)	47 (24.35)	3 (1.55)	**1.74**	1.14
5	… were they helped to make a treatment plan that they could carry out in their daily life.	70 (36.34)	13 (6.74)	11 (5.70)	20 (10.36)	38 (19.7)	39 (20.21)	2 (1.04)	**2.63**	1.52
6	… were they helped to plan ahead so they could take care of their condition even during hard times.	74 (38.3)	18 (9.33)	11 (5.70)	19 (9.84)	35 (18.13)	34 (17.62)	2 (1.04)	**2.51**	1.51
7	… were they asked how much their chronic disease affected their life.	51 (26.42)	19 (9.84)	32 (16.58)	27 (13.99)	39 (20.21)	25 (12.95)		**2.91**	1.45
8	… were they contacted after a visit to see how things were going.	77 (39.90)	28 (14.50)	29 (15.0)	16 (8.3)	30 (15.5)	10 (5.2)	3 (1.55)	**2.41**	1.46
9	… were they told how much visits with other types of doctors, like an eye doctor or surgeon, helped their treatment.	50 (25.91)	13 (6.74)	20 (10.36)	25 (12.95)	62 (32.12)	21 (10.88)	2 (1.04)	**3.21**	1.58
		**not at all satisfied** **n (%)**	**generally not satisfied** **n (%)**	**moderately satisfied** **n (%)**	**rather satisfied** **n (%)**	**completely satisfied** **n (%)**	**unable to rate** **n (%)**	**Missing** **n (%)**	**Mean rating**	**SD**
10	Overall, how satisfied was the decedent with the medical care received?	15 (7.77)	22 (11.40)	41 (21.24)	65 (33.68)	41 (21.24)	8 (4.15)	1 (0.52)	**3.52**	1.20

^a^Key for of answer categories: almost never = 1, generally not = 2, sometimes = 3, most of the time = 4, almost always = 5

**Fig. 1:**
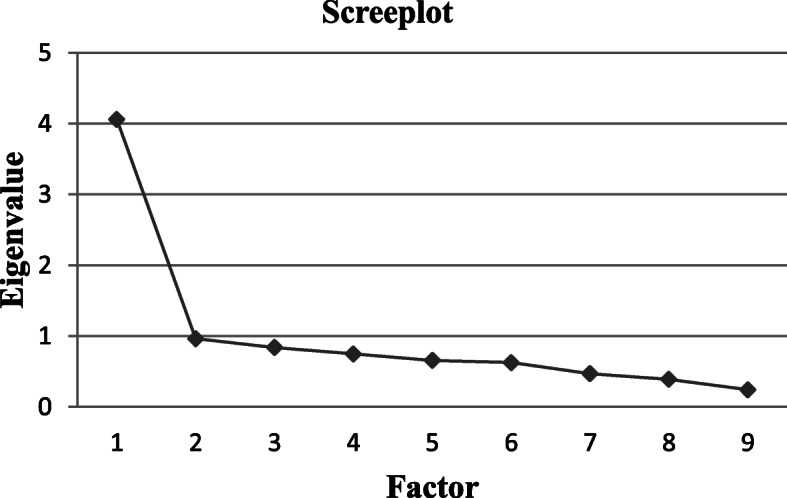
Screeplot of exploratory factor analysis (*N* = 193)

Table 3 displays the results of the factor analysis for the PACIC-S9-Proxy. A one-dimensional structure of the questionnaire was observed in the exploratory factor analysis where the Eigenvalue drops to 0.97 at the second factor (see Fig. 1).

**Table 3:** Results of factor analysis

Item	How did your relative experience the care for their chronic illness during the last year of life?	Factor loading	Kaiser-Meyer-Olkin
1	… were they given choices about treatment to think about.	0.66	0.92
2	… were they satisfied that their care was well organized.	0.62	0.91
3	… were they given a copy of their treatment plan.	0.58	0.93
4	… were they encouraged to get to a specific group or class to help them cope with their chronic condition.	0.46	0.92
5	… were they helped to make a treatment plan that they could carry out in their daily life.	0.74	0.81
6	… were they helped to plan ahead so they could take care of their condition even during hard times.	0.82	0.81
7	… were they asked how much their chronic disease affected their life.	0.76	0.90
8	… were they contacted after a visit to see how things were going.	0.57	0.80
9	… were they told how much visits with other types of doctors, like an eye doctor or surgeon, helped their treatment.	0.75	0.87

Overall, the analysis shows an explained variance of 45.12%, a KMO of 0.86, and a significant Barlett’s test of sphericity with *p* < 0.001. The internal consistency estimated using Cronbach’s alpha was 0.84. The factor loadings are displayed in Table 3 and ranged from 0.46 for item 4 to 0.82 for item 6.

A sensitivity analysis (not shown) including only those informants who completed all items of the PACIC-S9-Proxy (*N* = 94) revealed comparable results to those observed in the main analysis.

## Discussion

This study evaluated the validity of an adapted PACIC short form questionnaire for surveying proxy informants on the level of patient-centeredness experienced in care during the decedents’ LYOL. The one-factor structure of the original instrument was also observed in this analysis of the adapted version. The instrument can be rated as having good internal consistency with a Cronbach’s alpha of 0.84. Informants rated the item” given a copy of their treatment plan” highest, while “encouragement to get to a specific group or class to cope with the condition” was rated lowest. The highest and lowest rated items seem to reasonably represent care practices in Germany. Since 2016, patients who are receiving three or more types of medication for treatment are legally entitled to receive a structured and detailed medication plan (Section 31a Volume 5 of the German Social Code Book [SGB V] [29]. As patients in their LYOL are often multi-morbid and therefore usually fulfil the criterion of receiving three or more types of medication [25], it is plausible that this item reflects the most frequently implemented aspect of PCC. “Encouragement to get to a specific group or class to cope with the condition” might have been the lowest rated item due to healthcare providers considering the decedents physically, mentally or emotionally incapable of participating in such groups.

In addition to the medication plan, there is also a legal basis requiring structured care management in relation to transitions from hospitals to other care organizations, or to the patient’s home. Since October 2017, patients within the German social health insurance system are entitled to structured discharge management. Despite this, the item satisfaction with the organization of care was rated low. Since data collection only started in November 2017 the discharge management might not have been implemented to a high degree in all organizations. Moreover, each additional transition between healthcare settings, increases the chances of organizational problems in the transitions. Since transitions from one care organization to another are common in the LYOL, this might explain the low degree of satisfaction. Other studies from the perspective of informal caregivers have also observed problems in discharge management and unorganized transitions [30]. Future surveys using the same instrument may observe improvements in discharge management. None of the other items are addressed specifically in laws or regulations, so their level of implementation is therefore up to the individual providers.

As with the widely applied original long version of the PACIC we assume that, if translated appropriately, the PACIC-S9-Proxy can be applied in other countries [31, 32]. The concept of PCC is accepted internationally and many healthcare systems and providers strive for its implementation, especially in Europe and Northern America. As such, the items can also be considered relevant to other countries. Nevertheless, we expect scores for individual items to differ across countries due to different regulations, processes or traditions in healthcare provision, such as obligatory medication plans or availability of palliative care services.

### Strengths and limitations

The broad recruitment strategy and inclusion of informants with different relationships to the decedents is a strength of this study, as is it reporting on decedents with a diverse background in disease and treatment experiences. This allows us to gain a broad overview of the care process in the LYOL instead of looking at only one healthcare provider. Another strength is the inclusion of the answer category “unable to rate” in the instrument. Including this item enabled participants to judge their ability and knowledge to serve as a proxy on the respective items, instead of being forced to provide guessed answers or producing missing values for which reasons are not traceable. It allowed for the prevention of random answer behavior and led to less than 1% missing values. The overall data quality can therefore be rated as high. Our study has several limitations. Firstly, participants were recruited mainly via self-selection, which might have biased the sample, as the informants that completed the survey were thusparticularly active and involved. However, direct recruitment via practice partners is assumed to minimize this potential bias. Second, this study’s methods did not include a comparison with patient judgements of PCC. However, previous studies have already shown agreement in relation to quality of services [19], of which patient-centeredness can be one aspect. We are not able to assess any potential bias introduced by surveying proxies instead of self-report answers.

## Conclusions

The adapted PACIC-short form as a proxy version is suitable for eliciting the level of patient-centeredness in the care during the LYOL. The instrument might facilitate improvement of care during the LYOL by identifying areas in which patients or informants report low levels of patient-centeredness. The agreement between the patients’ and the proxy informants’ perceptions of patient-centeredness should be addressed in future studies, in samples where comparison between patient and proxy judgements might be possible.

## Supplementary Information

**Additional file 1: Supplementary file 1.** English Version: Patient Assessment of Chronic Illness Care Short Form for Proxies (PACIC-S9-Proxy)

## Data Availability

The datasets generated and/or analyzed during the current study are not publicly available due to data protection regulations, but are available from the corresponding author upon reasonable request and following application to CoRe-Net (Core-Net@uk-koeln.de).

## Abbreviations

IOM: Institute of Medicine
KMO: Kaiser-Meyer-Olkin
PACIC-S9-Proxy: Patient Assessment of Chronic Illness Care- short form containing nine items for completion by proxy informants
PCC: Patient-centered care
