# Supplementary material for: Patient-centered care during the last year of life: adaptation and validation of the German PACIC short form for bereaved persons as proxies (PACIC-S9-proxy)
Source: BMC Palliat Care. 2020 Nov 24;19:177. doi: 10.1186/s12904-020-00687-x (PMC7687735; doi:10.1186/s12904-020-00687-x)
Supplement: Supplementary file 1 — Additional file 1: Supplementary file 1. English Version: Patient Assessment of Chronic Illness Care Short Form for Proxies (PACIC-S9-Proxy) [file 12904_2020_687_MOESM1_ESM.pdf]

Supplementary file 1
English Version: Patient Assessment of Chronic Illness Care Short Form for Proxies (PACIC-S9-Proxy)

**What was your relative's experience of the care they received for their chronic illness during the last year of life?**						
**During the last year of life…**	**almost never**	**generally not**	**sometimes**	**most of the time**	**almost always**	**unable to rate**
…were they given choices about treatment to think about.	☐	☐	☐	☐	☐	☐
… were they satisfied that their care was well organized.	☐	☐	☐	☐	☐	☐
…were they given a copy of their treatment plan.	☐	☐	☐	☐	☐	☐
…were they encouraged to get to a specific group or class to help them cope with their chronic condition.	☐	☐	☐	☐	☐	☐
…were they helped to make a treatment plan that they could carry out in their daily life.	☐	☐	☐	☐	☐	☐
…were they helped to plan ahead so they could take care of their condition even during hard times.	☐	☐	☐	☐	☐	☐
…were they asked how much their chronic disease affected their life.	☐	☐	☐	☐	☐	☐
…were they contacted after a visit to see how things were going.	☐	☐	☐	☐	☐	☐
…were they told how much visits with other types of doctors, like an eye doctor or surgeon, helped their treatment.	☐	☐	☐	☐	☐	☐
	**not at all satisfied**	**generally not satisfied**	**moderately satisfied**	**rather satisfied**	**completely satisfied**	**unable to rate**
Overall, how satisfied was the decedent with the medical care received?	☐	☐	☐	☐	☐	☐
